# Justification of Sentencing Decisions: Development of a Ratio-Based Measure Tested on Child Neglect Cases

**DOI:** 10.3389/fpsyg.2021.761536

**Published:** 2022-01-14

**Authors:** Eiichiro Watamura, Tomohiro Ioku, Toshihiro Wakebe

**Affiliations:** ^1^Graduate School of Human Sciences, Osaka University, Suita, Japan; ^2^Division of Psychology, Department of Human Sciences, Seinan Gakuin University, Fukuoka, Japan

**Keywords:** sentencing decision, punishment, retribution, rehabilitation, justification, sentencing criteria, judicial sentence severity

## Abstract

Theoretically, people’s justification of a sentencing decision involves a hybrid structure comprising retribution, incapacitation, general deterrence, and rehabilitation. In this study, a new ratio-type measure was developed to assess this structure and was tested to detect changes in the weighting of justification according to the content emphasized in a particular crime. Two child neglect scenarios were presented to participants, where they read either a severe-damage scenario (where a single mother’s selfish neglect caused her son’s death) or a moderate-damage scenario (where a single mother became apathetic due to economic deprivation and caused her child’s debilitation). Participants then indicated the proportion of importance they placed on each justification in determining the defendant’s punishment, with an overall proportion of 100%, along with responding to the sentence on an 11-point scale. This study involved a two-factor analysis of variance for justification ratios, a *t*-test for the sentence, and a multiple regression analysis with three demographic variables, the four justifications as independent variables, and the sentence as the dependent variable. The ratio of retribution to rehabilitation was reversed depending on the scenario: in the severe-damage scenario, retribution was weighted highest at 27.0% and rehabilitation was weighted at only 19.0%. By contrast, in the moderate-damage scenario, rehabilitation had the highest weighting of about 26.2%, while retribution was weighted at 21.5%. The sentence was more severe in the severe-damage scenario. Multiple regression analysis suggested that in the severe-damage scenario, most participants failed to deviate from choosing retribution by default and decided on heavier sentences, while some who considered rehabilitation and incapacitation opted for lighter sentences. The present measure succeeded in detecting changes in the weighting of justification, which can be difficult to detect with common Likert Scales. In addition, it was found that not only retribution but utilitarian justification was considered in the sentencing decisions of serious cases.

## Introduction

Sentencing justification is based on a hybrid of four categories: retribution, incapacitation, general deterrence, and rehabilitation (Robinson, 1987; Exum, 2017; Hoskins, 2020). Retribution can be considered as a past-oriented form of justification, whereby the offender is given a punishment that is commensurate with the severity of the crime, thereby correcting a moral imbalance (Carlsmith, 2006). Therefore, according to the retribution approach, punishment should be proportionate to the severity of the crime. Utilitarian justification, on the other hand, is a future-oriented, pragmatic perspective aiming to deter future crimes by influencing the criminals themselves or society in general (e.g., Vidmar and Miller, 1980; Weiner et al., 1997; Carlsmith, 2006). Utilitarian justifications can be categorized into incapacitation, general deterrence, and rehabilitation (or education) (McFatter, 1978; Robinson, 1987; McMunigal, 1998). Incapacitation is based on the assumption that the cause of crime is inherent in the offender and attempts to deter future crimes by isolating criminals from society for a certain period of time, such as by imposing long prison sentences for dangerous offenders (Goldman, 1982). General deterrence means that laypeople should be deterred from becoming potential criminals by showing them that they will be punished as severely as possible if they break the law (Nagin, 1998). When the incidence of crime is high, and the arrest rate is low, the need to make an example of the offender is high, and sentences for those who have been taken into custody become more severe. Rehabilitation seeks to deter future crime by working directly with offenders. This is more favorable to criminals than the other aforementioned approaches to justification; rehabilitation aims to reduce criminal intent and ultimately transform a person into a law-abiding citizen who can contribute to society (Robinson, 1987; Cotton, 2000). Although these four types are not the only sentencing goals possible for punishment, they are the most commonly endorsed by the public in the justice system (e.g., McFatter, 1978; Cotton, 2000) and have been adopted in several empirical studies examining sentencing decisions of the general population (e.g., Roberts and Gebotys, 1989; Templeton and Hartnagel, 2012; Niang et al., 2020).

Previous empirical studies have reported that the public adopts retribution as the predominant or nearly sole justification (McCorkle, 1993; Weiner et al., 1997; Carlsmith et al., 2002; Oswald et al., 2002; Orth, 2003; Rucker et al., 2004; Carlsmith, 2006, 2008; Alter et al., 2007; Carlsmith and Darley, 2008; Gromet and Darley, 2009; Okimoto et al., 2009; Keller et al., 2010; Watamura et al., 2011; Gerber and Jackson, 2013; Twardawski et al., 2020). With respect to sentencing decisions for serious crimes such as murder, retribution is the default justification “…their natural (default) approach to sentencing probably involved retribution” (Carlsmith, 2006, p. 447). However, the predominance of retribution does not imply that the other justifications are not considered. Moreover, retribution and other justifications are not necessarily conflicting (Crockett et al., 2014). For instance, long imprisonment may offset serious harm (retribution) and through punishment, restore justice that would otherwise be lost to crime. At the same time, it may support the transformation of offenders into citizens that disengage from crime (rehabilitation), withhold them from opportunities to reoffend (incapacitation), and intimidate potential offenders (general deterrence).

Prior studies have been limited in their ability to capture the relative weight people assign to the four justifications. With some exceptions (e.g., O’Toole and Fondacaro, 2017), previous studies have involved participants rating each of the four justifications for sentencing separately (e.g., Krosnick, 1999; McKee and Feather, 2008; Berryessa, 2018). Thus, the purpose of this study was to develop a measure that can assess mixed justifications of punishment. Furthermore, the new measure developed as part of this study was tested on the same type of offense to detect changes in the weighting of justification according to the emphasized content.

According to the hybrid theory, people do not determine punishment by retribution alone. As an individual characteristic, the tendency to blame and seek retribution against offenders is positively correlated (0.70) with permissive utilitarianism, which considers inflicting severe punishment as a means of deterrence (Yamamoto and Maeder, 2021). An experiment with college students found that as the length of incarceration increased, punishment appropriateness ratings increased, and participants were also more positive about the acceptance of offender rehabilitation (Brubacher, 2019). This suggests that participants perceived incarceration to be effective for both retribution and rehabilitation. In a scenario experiment conducted with members from the general population (Spiranovic et al., 2012), the most common justification chosen for sentencing serious crimes was a mixture of retribution and utilitarianism (burglary 36.0%, assault 34.4%); a single justification, including retribution, was less commonly selected. In the trust game paradigm (Cañadas et al., 2015), in which both parties maximize their mutual benefit by repeating the process of returning some or all of the money entrusted by the other party without monopolizing the money, the experimental manipulation of whether or not the punishment is accompanied by the message “I have punished you” can more clearly identify the justification of punishment. If the message is not conveyed to the punished party, the punishment is only self-satisfying and will not deter the next betrayal. In a study by Crockett et al. (2014), participants who played the role of the punisher were motivated to reduce the amount of money distributed to participants who acted as violators (i.e., punish them) by two types of justification: retribution, which without the message, seeks to punish violators based on mere moral revulsion (not related to the possibility of deterrence), and utilitarian justification, which with the message, seeks to deter violations of the distribution rule through punishment. In other words, in the present-message condition in this study, retribution and deterrence justifications are mixed. Thus, the general population determines the output punishment by changing the weighting of any of these justifications (i.e., by assigning more or less weight).

Unfortunately, assessment of the hybrid structure of sentencing justification is currently limited because of challenges in inferring the exact ratio. The most popular way to measure justification is through a unipolar Likert Scale. In this scale—for the items that ask, “how important is this justification?”—the scores are moderately or more highly aligned for almost all responses (i.e., acquiescence-response bias; Krosnick, 1999). For example, in McKee and Feather’s (2008) seven-point scale, the overall sentencing decisions for criminal offenders were retribution (*M* = 4.66, *SD* = 1.16), incapacitation (*M* = 4.49, *SD* = 0.99), general deterrence (*M* = 4.74, *SD* = 1.06), and rehabilitation (*M* = 4.28, *SD* = 1.20). In Berryessa’s (2018) study, which asked participants to respond with a finer scale ranging from 1 to 100, the scores for homicide related to the four justifications were: retribution (*M* = 68.83, *SD* = 30.03), incapacitation (*M* = 77.41, *SD* = 24.21), general deterrence (*M* = 77.85, *SD* = 26.02), and rehabilitation (*M* = 62.06, *SD* = 34.17). Similar trends were observed for the scenario of rape on the scores of retribution (*M* = 69.30, *SD* = 28.71), incapacitation (*M* = 75.24, *SD* = 27.21), general deterrence (*M* = 70.29, *SD* = 23.32), and rehabilitation (*M* = 63.56, *SD* = 31.70). Even with the fine-tuning of scores, most offenses had medium to high scores for all justifications. Some studies, wary of this lopsided distribution of scores, have used the scale in a manner that forced a trade-off between retribution and another justification. O’Toole and Fondacaro (2017) used the item “Relative to giving a young offender what he deserves, how important is it to you that the juvenile justice system improve the young offender’s psychological well-being?” to measure relative support for rehabilitation vs. retribution. However, although this scale shows the relative ratios of the two, it fails to convey the weighting of all four justifications.

In such cases, how can we assess that hybrid structure in which the sentencing justifications trade-off against each other? One solution is to implement the theoretical concept of four hybrids (Robinson, 1987; Exum, 2017; Hoskins, 2020) with the Summation Model (Hollands and Spence, 1998). According to the Summation Model, an anchor that represents the whole (such as 100%) makes it easier to perform the percentage judgment task intuitively. Therefore, a measure was devised in which the entire sentencing purpose was set to 100%, and the weighting of each justification was given a numerical input. For the description of the four justifications, Berryessa’s (2018) items were used for clarity and brevity (p. 245).

Since the Japanese judicial system is similar to the jury system in Germany and other European countries, the four types of justification have been examined in previous studies (e.g., Gollwitzer and Bücklein, 2007). Consistent with European and American study findings, the general Japanese public demonstrates the strongest preference for retribution (Kita and Johnson, 2014). Japan is the only industrialized country, other than the United States, to have the death penalty; its support for the death penalty varies from survey to survey, but it is high, at over 60%, due to the high support for notions of retribution, such as “life should be paid for with life” (Jiang et al., 2010b; Andreescu and Hughes, 2020). Despite the shared dominance of retribution in Japan with Europe and the United States, as a collectivist culture (Kitayama et al., 2009), Japan possesses the unique feature of strong social norms that seek adherence to social values by punishing perpetrators. Thus, Japan also demonstrates high support for general deterrence. In fact, studies comparing Japan with the United States have consistently reported stronger support for general deterrence in Japan (Gollwitzer and Bücklein, 2007; Jiang et al., 2010a). However, the degree of support toward general deterrence among Japanese people is not, in fact, clear; neither is its relative weightage in terms of other justifications, including retribution. Accordingly, the new measure developed in this study may help clarify the unique Japanese cultural characteristics. In the current study, we examined the hybrid structure that determines people’s sentencing decisions in Japan, where both retribution and general deterrence are dominant.

## Materials and Methods

### Overview

In this study, we examined whether the weighting of justification changed between a severe-damage scenario, where the damage was severe, and the offender’s rehabilitation potential was low, and its opposite, a moderate-damage scenario, where the damage was moderate, and the offender’s rehabilitation potential was high. We also examined the effect of the difference in weighting justification on the sentence. It was predicted that the ratio of retribution would be higher in the severe-damage scenario, resulting in a severer sentence, while the ratio of rehabilitation would be higher in the moderate-damage scenario, as the defendant would have a higher chance of being rehabilitated in contexts of less damage. Prior to the main study, a preliminary survey was conducted, and two scenarios were finalized for inclusion in the study.

### Preliminary Survey

In this experiment, where the new measure was tested for the first time, child neglect, a type of child abuse, was selected as the offense type over common violent offenses, as the offense needed to be manipulated for the severity and rehabilitation potential to be more pronounced. While the effect of the defendant’s experience of child abuse on justification has been studied (Berryessa, 2018, 2021), the effect of justification on sentencing decisions for child abuse also needs to be examined to break the negative cycle that leads to subsequent abuse. In Japan, where this study was conducted, public attention to child neglect deaths has increased immensely because of increased media coverage of recent fatal incidents and warnings from media experts (Takikawa, 2019). As a result, there are growing demands for harsher punishments for convicted parents. The fact that the crime was committed by the person who should protect the child heightens the sense of moral seriousness. It was postulated that if the crime was committed by a single mother with a compelling motive, such as poverty, the defendant would be seen as more likely to be rehabilitated. In the main study, it seemed important to compare scenarios with completely different sentences to determine the output of justifications. A preliminary survey was conducted online with participants from the general public (*N* = 135, female = 68, male = 67, *M*_*age*_ = 50.68, *SD* = 12.43), recruited from a Japanese internet research company. The participants read one of four scenarios combining two levels of damage (severe/moderate) and two levels of rehabilitation potential (low/high) (between-participants design) and judged the sentence for the defendant on the same scale as the main study (11-point scale, see below). The results showed that only one scenario, namely, moderate-damage and high potential, had the lowest sentence score (*M* = 6.63, *SD* = 2.67, *p* < 0.01). The main study’s purpose of comparing justification ratios cannot be achieved unless a comparison is made between scenarios with different sentences as the output. However, since the other three scenarios were almost identical (*M* = 7.97–8.46, *SD* = 1.65–2.82), suggesting that it is also difficult to separate the two factors, we decided to use the severe-damage and low potential scenario (*M* = 8.41, *SD* = 2.30) as representative of the three and compared the same with the only scenario with a different sentence. As explained earlier, these two scenarios are “the severe-damage scenario” and “the moderate-damage scenario.” The study was conducted online after obtaining ethical review approval from Osaka University in accordance with the guidelines of the Japanese Psychological Association. All data have been published on the Open Science Framework platform.^1^

### Power Analysis

As the required effect size of the multiple regression analysis was unknown, it was assumed to be 0.15 based on the *f*^2^ index suggested by Cohen (1988). The α was set at 0.05, the power of the test (1-β) was set at 0.80, and a power analysis was conducted in G*Power 3.1.9.7 (The G*Power Team, Heinrich Heine Universität) for a multiple regression analysis with seven predictors (four justifications plus three demographic variables). This analysis revealed that the required sample size was *N* = 103; hence, data were collected with a target of 103 for each scenario. In power analysis for other statistical tests, a much smaller required sample size was calculated. However, that would not have been adequate for the multiple regression analysis. Therefore, the final number of participants was determined based on the results of the power analysis for the multiple regression analysis.

### Participants

A total of 264 participants were recruited from a panel of individuals aged 20+ years who were registered with a Japanese internet research company. They resided in 42 prefectures in Japan and were representative of lay judges. They provided information on gender, age, parental status, marriage, prefecture, and their job as demographic variables at the time of participation. With the exception of age, these variables were coded to dummy variables such as 0, 1, 2. The reward for participation was points (equivalent to about 20 cents) that could be exchanged for an Amazon gift card. Those who could not provide informed consent (8) and those who did not respond to the questions about the defendant or provided unintelligible responses (discussed later in the procedure) (31) were excluded from the study. As a result, the sample size for the analysis was 112 (female = 56, male = 56) participants in the severe-damage scenario (*M*_*age*_ = 44.09, *SD* = 14.39) and 113 (female = 57, male = 56) participants in the moderate-damage scenario (*M*_*age*_ = 0.44.45, *SD* = 15.36). When the two groups were compared to examine differences in demographic variables, no significant differences were observed [gender: χ^2^(1) = 0.004, *p* = 0.947, age: *t*(223) = 0.182, *p* = 0.855, parental status: χ^2^(1) = 1.957, *p* = 0.162, marriage: χ^2^(1) = 0.213, *p* = 0.644, prefecture: χ^2^(41) = 47.610, *p* = 0.222, job: χ^2^(10) = 8.244, *p* = 0.605], and it was concluded that they were comparable in the subsequent analysis. The three variables of gender, age, and parental status were used as independent variables in the multiple regression analysis, along with the four justifications.

### Experimental Design

The current experiment comprised five blocks: Scenario reading, attention control task, in-house newly developed questionnaire, sentencing decision, and scenario manipulation check block.

First, participants read a child neglect case scenario of approximately 230 words (Supplementary Data). There were two types of scenarios, and participants were randomly assigned either one.

#### Severe-Damage Scenario

A single mother neglected and starved her 2-year-old child for more than 6 days to spend time with her boyfriend.

#### Moderate-Damage Scenario

A single mother neglected her 2-year-old child for more than 30 h due to economic deprivation and loss of energy, and the child wasted away.

The points emphasized by participants changed depending on the scenario. In the severe-damage scenario, the severity of the child’s death and the low rehabilitation potential derived from selfish motives were emphasized. By contrast, in the moderate-damage scenario, the child was in a harmful but not life-threatening situation, and the motive of poverty suggests a higher probability of rehabilitation.

After reading the scenario, in the second block, participants were asked to imagine themselves in the courtroom and write one question to the defendant. This question was a device used to make them read the scenario carefully, and the description was not analyzed.

Those who did not respond, or wrote an invalid question such as meaningless strings or “nothing” were excluded from the study, as they may not have read the scenario carefully. In the third block, participants responded to the new measure developed in this research, in which they were asked to enter positive integers to indicate the percentages of importance they placed on each of the four justifications: retribution, incapacitation, deterrence, and rehabilitation, as defined by Berryessa (2018). Notably, the present study considered the addition of another device, a dummy “precedent,” that is not theoretically related to the four justifications, so that the sum of the five items including the dummy would be 100%. Without the dummy, increasing the ratio of any of the justifications will decrease the ratios of the others. In other words, the independence of the observed values cannot be satisfied, and multicollinearity is strong when there is such a relationship between the measures in the analysis. However, when the dummy is included, “100—dummy” becomes the sum of the four justifications, and such a relationship is not necessarily established. As a result, the violation of independence of observed values is eliminated to some extent, and multicollinearity is mitigated. The new measurement had the following question and items: In deciding the punishment for this mother, how important are the following five items to you? Please assign a percentage to each such that the total is 100%. Items were presented randomly.

#### Retribution

Retribution relies on the idea that for justice to be served, an offender deserves to be punished in a manner that is proportionate to the severity and moral heinousness of the committed crime.

#### Incapacitation

Incapacitation aims to remove offenders from society to protect the public from future unlawful behavior.

#### General Deterrence

Deterrence attempts to prevent the future committal of crimes through the threat of future punishments that outweigh an individual’s motivation to commit future criminal acts.

#### Rehabilitation

Rehabilitation seeks ways to actively reform and address the underlying reasons for an offender’s criminal behavior so that an individual will not reoffend.

#### Precedent (Dummy)

The sentence should be determined based on the sentencing decisions handed down in previous abuse cases and judges’ opinions.

Next, in the fourth block, participants responded to the item “Please choose the one that is closest to your idea of punishment for this mother,” with the following 11 possible punishments for the defendant (Robinson and Darley, 1995; Brubacher, 2019): (1) No punishment, (2) 1 day in prison, (3) 2 weeks in prison, (4) 2 months in prison, (5) 6 months in prison, (6) 1 year in prison, (7) 3 years in prison, (8) 7 years in prison, (9) 15 years in prison, (10) 30 years in prison, and (11) life in prison. This particular question was designed to examine the difference in the severity of the sentence between the scenarios and the effect of justification on sentencing.

Finally, the participants responded to items to check the manipulation of the scenario. If the participants in the severe-damage scenario estimated the damage as severe, they would weight retribution. On the other hand, if the participants in the moderate-damage scenario estimated the possibility of the defendant’s rehabilitation highly, they would weight rehabilitation. Note that the purpose of this block was not to determine which justifications increased but to make sure that the factors in the scenario that increased justification (i.e., the child’s suffering, the mother’s potential for rehabilitation) were considered by the participants. For this purpose, the items needed to include the person in the scenario, such as the mother or the child; thus, the present study used a tentative modification of items from Weiner et al. (1997), which includes descriptions of specific persons. They rated the options on a 6-point scale ranging from “1: Do not at all agree” to “6: Very much agree.” The four items, one for each of the four justifications, were as follows:

1. Compared to other serious cases, the pain the child has suffered is much worse.
2. In order to prevent the mother from making the same mistake, it is important to keep her out of society.
3. I cannot help but wonder if this kind of child abuse is happening more often.
4. It is not entirely impossible that the mother can be rehabilitated.

### Statistical Analysis

In this study, four statistical analyses were conducted. Based on the percentages obtained through the new measure, averaged ratios were calculated for each justification by summing the percentages assigned to each justification (e.g., 30% for retribution) and dividing it by the number of participants in the scenario. The averaged ratios were compared by a two-factor analysis of variance (ANOVA) to determine the differences in scores between groups on each justification variable. Precedent, the dummy justification, was not examined here and later. In the second analysis, the means of the 11 magnitude levels were compared by a *t*-test to examine if there was a difference in sentencing severity between the two scenarios. Then, in the third analysis, multiple regression analysis was implemented to determine predictive relations between the four justifications, age, sex, and parental status as the independent variables and the magnitude of sentencing scores as the dependent variable. Prior to the multiple regression analysis, these dependent variables were standardized to have a mean of 0 and standard deviation of 1 by z-score normalization, as per previous studies (Weiner et al., 1997; Okimoto et al., 2009), because they differed in units due to being coded and converted to ratios. Moreover, we checked the assumptions of regression analysis by examining normality according to whether the residuals followed a normal probability-probability plot, homogeneity of variance according to whether the residuals were dispersed, and independence of observed values from the correlation coefficients. For the four manipulation check items measured by the 6-point scale, the mean scores of the child’s suffering, which strengthens retribution, and the mother’s rehabilitative potential, which strengthens rehabilitation, were calculated for each scenario and compared by *t*-test (we also compared the factors that increased incapacitation and general deterrence to confirm that there was no difference). All analyses were performed using the HAD (Shimizu, 2016), a statistical software program that can analyze Excel format data with high accuracy.

## Results

### Justification

**FIGURE 1:**
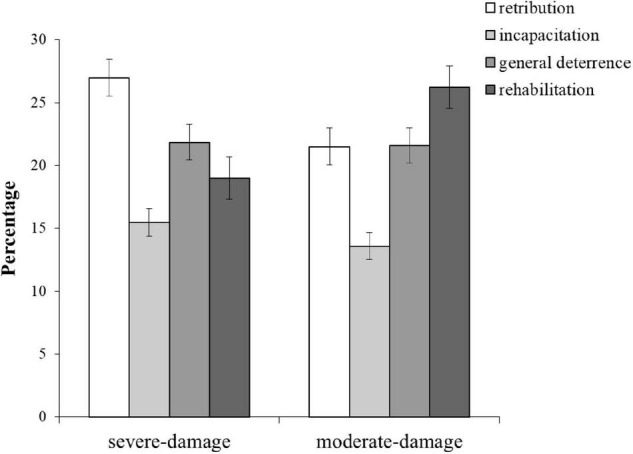
Percentage of each justification when the total is 100%. The error bars indicate the standard error.

The bivariate correlations for justifications are shown in Table 1, and the mean ratio for each justification is shown in Figure 1. Note that the ratio of retribution to rehabilitation is completely reversed in each scenario. In the severe damage scenario, retribution and incapacitation showed significant negative correlations with general deterrence and rehabilitation, respectively (*r*s < –0.258, *p*s < 0.01). A slightly weaker negative correlation between general deterrence and rehabilitation was also shown (*r* = –0.185, *p* < 0.10). In the moderate-damage scenario, retribution showed a significant positive correlation with incapacitation (*r* = 0.202, *p* < 0.05) and negative correlations with general deterrence and rehabilitation (*r*s = –0.280, *p*s < 0.01). Moreover, incapacitation and general deterrence showed significant negative correlations with rehabilitation (*r*s < –0.372, *p*s < 0.01). A two-factor ANOVA for the mixed design with scenario and justification as independent variables revealed significant differences in the interaction effect [*F*(3, 669) = 5.448, *p* = 0.002; partial η*_*p*_*^2^ = 0.024] and the main effect of justification [*F*(3, 669) = 14.024, *p* = 0.000; partial η*_*p*_*^2^ = 0.059]. Multiple comparisons using the Holm method confirmed the prediction, and the test was successful. In the severe-damage scenario, retribution was the highest, accounting for 27.0%, significantly higher (*p* = 0.015, *d* = 0.474) than rehabilitation (19.0%) and with a difference of 8%. By contrast, in the moderate-damage scenario, rehabilitation was the highest (26.2%) and differed from retribution (21.5%) by approximately 5%, which was not significant. Multiple comparisons using Tukey’s HSD method confirmed this inverse relationship; in the severe damage scenario, retribution was significantly higher than rehabilitation (*p* < 0.001), and in the moderate-damage scenario, rehabilitation was significantly higher than retribution (*p* = 0.014). To summarize, as predicted, the weighting of justification changed depending on the scenario, and the new measure confirmed that the ratio of retribution to rehabilitation was reversed.

**TABLE 1:** Bivariate correlations of justifications.

		Severe-damage scenario					Moderate-damage scenario				
		*M*	*SD*	1	2	3	*M*	*SD*	1	2	3
1	Retribution	26.973	18.298				21.504	12.154			
2	Incapacitation	15.482	13.064	–0.105			13.593	9.471	0.202*		
3	General deterrence	21.839	16.425	−0.316**	−0.258**		21.584	13.426	−0.280**	–0.066	
4	Rehabilitation	19.009	15.464	−0.495**	−0.352**	−0.185^+^	26.239	20.039	−0.519**	−0.541**	−0.372**

**p < 0.01, *p < 0.05, ^+^p < 0.10.

Although not the focus of this study, incapacitation was found to have the lowest ratio in both scenarios. In the severe-damage scenario, incapacitation was significantly lower for both retribution and general deterrence (*p* = < 0.001, *d* = 1.125; *p* = 0.006, *d* = –0.476, respectively). In the moderate-damage scenario, the difference between incapacitation and rehabilitation, general deterrence, and retribution were all significant (*p* < 0.001, *d* = –0.840; *p* < 0.001, *d* = –0.598; *p* < 0.001, *d* = –0.579). As suggested as a Japanese cultural characteristic, general deterrence was consistently rated second highest, regardless of the scenario (severe-damage 21.8% vs. moderate-damage 21.6%).

### Severity of Sentencing

A *t*-test was conducted to determine whether there was a difference in the sentence severity, as measured by the 11-point scale (Robinson and Darley, 1995; Brubacher, 2019) after the justification ratio-based measure, between the scenarios: the severe-damage scenario had a mean of 8.50 (*SD* = 1.95), and the moderate-damage scenario had a mean of 6.92 (*SD* = 2.31), confirming that the severe-damage scenario resulted in significantly heavier sentences, [*t*(223) = 5.533, *p* < 0.001, *d* = 0.735].

### Effect of Justification on Sentencing

Next, multiple regression analysis was conducted for each scenario with sentence severity as the dependent variable and gender, age, parental status, and the four justifications as independent variables. The results are shown in Tables 2, 3. In the moderate-damage scenario, a higher ratio of rehabilitation tended to result in a lighter sentence (β = –0.257); however, the standard partial regression coefficients for all variables were non-significant. By contrast, in the severe-damage scenario, a higher ratio of incapacitation and rehabilitation was associated with a lighter sentence (β = –0.450, *p* = 0.002 and β = –0.314, *p* = 0.011, respectively). In both scenarios, demographic variables did not predict the sentence.

**TABLE 2:** Regression analysis (severe-damage scenario).

	β	*p*	95% CI		VIF
Sex	0.045	0.593	–0.123	0.214	1.102
Age	–0.131	0.157	–0.312	0.051	1.281
Parental status	–0.070	0.439	–0.248	0.109	1.237
Retribution	0.114	0.406	–0.157	0.385	2.862
Incapacitation	−0.314*	0.011	–0.554	–0.075	2.232
General deterrence	0.116	0.337	–0.123	0.354	2.211
Rehabilitation	−0.450**	0.002	–0.733	–0.167	3.106
*R* ^2^	0.320	**			
Adjust *R*^2^	0.274	**			

*F(7, 104) = 6.979, p < 0.001, AIC = 441.813, BIC = 466.279.*

**p < 0.01, *p < 0.05, ^+^p < 0.10.

*Coefficients represent standardized coefficients, CI, confidence interval; VIF, Variance Inflation Factor; AIC, Akaike information criterion; BIC, Bayesian information criterion.*

**TABLE 3:** Regression analysis (moderate-damage scenario).

	β	*p*	95% CI		VIF
Sex	0.114	0.243	–0.078	0.306	1.274
Age	0.082^†^	0.457	–0.135	0.298	1.622
Parental status	–0.030	0.752	–0.221	0.160	1.252
Retribution	0.190	0.172	–0.084	0.465	2.603
Incapacitation	0.123	0.307	–0.115	0.360	1.947
General deterrence	0.063	0.644	–0.206	0.332	2.506
Rehabilitation	–0.257	0.154	–0.613	0.098	4.363
*R* ^2^	0.227	**			
Adjust *R*^2^	0.175	**			

*F(7, 105) = 4.401, p < 0.001, AIC = 497.941, BIC = 522.487.*

**p < 0.01, *p < 0.05, ^+^p < 0.10.

*Coefficients represent standardized coefficients, CI, confidence interval; VIF, Variance Inflation Factor; AIC, Akaike information criterion; BIC, Bayesian information criterion.*

### Scenario Manipulation Check

Based on a manipulation check item used to compare the mean ratings of the pain suffered by the child, the severe-damage scenario scored significantly higher than the moderate-damage scenario [4.68 (*SD* = 1.32) vs. 4.33 (*SD* = 1.26)], suggesting that the damage was considered more severe, [*t*(223) = 2.03, *p* = 0.044, and *d* = 0.532]. The mean rating of the defendant’s rehabilitative potential was not significant but was higher for the moderate-damage scenario [3.59 (*SD* = 1.28) vs. 3.81 (*SD* = 1.25)] *t*(223) = –1.28, *p* = 0.203, and *d* = –0.091. Differences in the scores of other items were also not significant [incapacitation: 3.29 (*SD* = 1.43) vs. 2.99 (*SD* = 1.33), *p* = 0.101; general deterrence: 4.37 (*SD* = 1.34) vs. 4.43 (*SD* = 1.29), *p* = 0.700].

## Discussion

### Availability and Theoretical Suitability of the Measure

As hypothesized, the results indicated that the weighting of justification changed according to the emphasized content, and the change could be detected by the new measure developed as part of this research. The results were completely symmetrical, with a higher rate of retribution observed in the severe-damage scenario and a higher rate of rehabilitation found in the moderate-damage scenario. Furthermore, it was found that all four justifications of retribution, incapacitation, general deterrence, and rehabilitation (although weighted differently) were considered in a certain ratio in the determination of punishment. Thus, the theoretical assumption of the four-hybrid structure of sentencing justification (Robinson, 1987; Exum, 2017; Hoskins, 2020) was supported. Furthermore, by showing that the weighting of retribution and rehabilitation was completely reversed in different scenarios, this study highlighted scenarios when retribution, considered the default justification, surrenders its place. Thus, the results suggest that the weighting of the hybrid structure can be flexible.

### Coexistence of Retribution and Utilitarian Justification

Prior research has consistently demonstrated the tendency among the general population to make sentencing decisions based on retribution (e.g., Weiner et al., 1997; Carlsmith, 2006; Keller et al., 2010; Gerber and Jackson, 2013; Twardawski et al., 2020). The results of this study also confirmed that the weighting of retribution in sentencing decisions in serious cases can be somewhat predominant but did not support retribution as the only justification. Since retribution is the default approach (Carlsmith, 2006), it is more likely to be weighted, but that does not negate utilitarian justification from consideration. In fact, even the severe-damage scenario, where the child was killed by the defendant who should have protected him, did not result in retribution alone. The finding that general deterrence was the second-highest weighted in both scenarios clearly indicates a Japanese tendency to emphasize general deterrence (Gollwitzer and Bücklein, 2007; Jiang et al., 2010a), suggesting that this measure could also reflect cultural characteristics, despite the similarity between Japan and the West in terms of the dominance of retribution for serious crimes. The consistent preference for general deterrence is indirect evidence of Japanese perceptions of punishment, such as a society that is more likely to maintain cohesion through applying severe punishments such as the death penalty (Johnson, 2020) and a tight culture with a low tolerance for deviant behavior (Gelfand et al., 2011). This finding is consistent with existing research results stating that punishment is motivated by both retribution and utilitarianism (Crockett et al., 2014) and that the mixed justification approach is most supported (Spiranovic et al., 2012). In addition, this study demonstrated the specific ratio of the mixed justification. Once the specific ratio is clarified, the difference in the weighting of justification might explain why the severity of the sentence varies depending on the cases and the judges.

### Advantages Over a Likert Scale

Almost all the scores on a Likert Scale will lie within the middle to high range (i.e., acquiescence-response bias; Krosnick, 1999), which makes it difficult to determine the importance of each item. Previous studies that examined the relationship between punishment and justification have been plagued by this problem. The measure developed in this study makes it possible to compare the four weightings. It is possible to test whether the factor loadings are different by using a Likert Scale, for example, by performing invariance tests between each scenario. Importantly, however, this ratio-type measure is effective even when justifications are conflicting. In this study, we manipulated damage levels and rehabilitation potential; thus, various combinations can emerge as there are multiple factors affecting justifications and sentencing decisions. Depending on the combination, it can become difficult to analyze loadings using Likert scale scores. For example, if a young person with multiple prior convictions commits a burglary, or an adult with no prior convictions commits a serious assault, the Likert Scale would yield similar scores for both retribution and rehabilitation (Spiranovic et al., 2012). As a result, both may appear to have been given equal weightage. However, in reality, people will always have to choose between maintaining the emphasis on retribution (as the default) or believing in the rehabilitative potential of the offender and emphasizing rehabilitation. The present measure can determine which aspect is given more weighting and loading during sentencing. Furthermore, it would be useful to examine not only individual cases but also attitudes toward the judicial system. In a study that examined the correlation between the death penalty and justification using a Likert Scale, it was found that the higher the weighting for retribution and general deterrence, the higher the support for the death penalty in the United States, Japan, and China (Jiang et al., 2010b). For such a study on the justice system, the present scale may provide a clearer picture of how people’s attitudes are determined based on any hybrid structure of justification.

### Prediction of Sentencing Decisions

The results of the multiple regression analysis showed that justification, which was the main factor in each scenario, did not predict the sentence severity; in the severe-damage scenario, the higher the ratio of incapacitation to rehabilitation, the lighter the sentence, while retribution did not predict the sentence. In the moderate-damage scenario, rehabilitation did not predict sentence severity. At first glance, these results seem to contradict the predictions. However, they may be rather consistent considering that retribution is considered the default approach (Carlsmith, 2006). In the severe-damage scenario, most participants might have been unable to deviate from the tendency to opt for retribution by default and thus increased the severity of their sentences, while some exceptional participants who emphasized rehabilitation and incapacitation could have lowered the severity of their sentences. The negative impact of incapacitation, which was not focused on in this study, on sentencing in the severe-damage scenario suggested that the risk of having another child and abusing that child is so low that a longer sentence may not be necessary. Even in the moderate-damage scenario, a certain weight was placed on retribution (21.5%), leading to a competition between rehabilitation and retribution and neither individually predicting the sentence. Thus, in both scenarios, the extent to which the weight of other justifications can be increased against the default weight of retribution was crucial to predict the sentence.

### Implications

Empirical data using this new measure as a “litmus test” can be applied to trial procedures. A test similar to the present study can identify jurors with extremely biased justifications in the selection process and detect the impact of specific evidence (e.g., gruesome evidence or victim impact statements) on jurors from changes in the justification ratio. The more jurors can visualize the balance that individuals place on justification, the easier it will be for them to work toward a consensus. If the balance between retribution and utilitarian justification is important in sentencing (as in many countries), this measure can help examine whether the public is actually making judgments in accordance with this principle and suggest necessary improvements. Recently, some studies have measured physiological indicators and psychological benefits to victims to understand restorative justice, which aims to repair the relationship between victims and offenders (e.g., Lloyd and Borrill, 2020; Witvliet et al., 2020). While there are some studies (e.g., Daly, 2002) that theoretically compare the similarity of restorative justice with retribution, to understand the concept of restorative justice, a new angle should be to examine the proportion of the hybrid structure of justification when restorative justice is supported more.

### Limitations and Future Research

The current study has some limitations. To test the new measure, cases of child neglect were deliberately chosen over general violent crimes. This was considered to manipulate and make the severity and rehabilitative potential of the cases more pronounced. Therefore, further research needs to verify whether the measure can detect changes in other cases that fall within the purview of the criminal justice system as well. While they were combined as a set in this test, crime severity and predictors of rehabilitation (i.e., selfishness and poverty) could have been manipulated separately in a 2 × 2 design to examine the effect of rehabilitation on sentencing decisions. In addition, it is necessary to examine the possibility of detecting changes in justifications other than retribution and rehabilitation. Based on Carlsmith’s (2006) discussion, the weighting of general deterrence may change with the manipulation of the frequency or detection rate of the crime, and that of incapacitation may change with the manipulation of the likelihood of defendant violence. Thus, it will be necessary to explore the applicability of the new measure by manipulating various factors separately and using a variety of cases. This measure should be widely tested outside Japan. The findings that the difference between retribution and rehabilitation was not significant in the moderate-damage scenario and that retribution did not predict the sentence may have been related to the cultural characteristics of the Japanese sample, which emphasizes general deterrence. It would be useful to understand the basic principles of the theory of punishment by examining how much the ratio of retribution, “the default,” and the ranking of other justifications are common across cultures and countries. Furthermore, with regards to collating responses, it may be more effective to use the method of dragging and moving a slider bar rather than the approach of entering numerical values as used in this study. The Summation Model (Hollands and Spence, 1998) was followed to implement the theoretical concept of the four hybrid constructs in the new measure. If the slider bar is a better fit to this model, which we assume may lead to an improvement, participants will be more likely to respond, and their responses will more closely reflect their sentencing justification. This study reflected high multicollinearity because there was only one dummy in the current study (i.e., precedent; see Tables 2, 3). Accordingly, it would be effective to include multiple dummies to mitigate multicollinearity, which would be easier using a slider bar.

## Conclusion

Sentencing justification among the public follows the hybrid structure of retribution, incapacitation, general deterrence, and rehabilitation. In the present study, a ratio-type measure was developed to access this structure, and its usefulness was tested on a single type of crime. The study succeeded in detecting changes in the weighting of justification, which was previously not detected by the existing form of assessment involving the Likert Scale. In addition to the finding of previous studies that retribution is the most important justification in sentencing decisions, the present study found that retribution is not the only justification and that other justifications are also considered—although retribution is more likely to be weighted as the default.

## Data Availability Statement

The datasets presented in this study can be found in online repositories. The names of the repository/repositories and accession number(s) can be found below: https://osf.io/n3a6s/.

## Ethics Statement

The studies involving human participants were reviewed and approved by the Ethical Review Committee for Behavioral Sciences, Graduate School of Human Sciences, Osaka University. The patients/participants provided their written informed consent to participate in this study.

## Author Contributions

EW conducted the material preparation and data collection, performed the analysis, and wrote the first draft of the manuscript. EW and TI conducted the analysis and developed the discussion. TI and TW commented on previous versions of the manuscript and made modifications. All authors contributed to the study conception and design, read and approved the final manuscript.

## Conflict of Interest

The authors declare that the research was conducted in the absence of any commercial or financial relationships that could be construed as a potential conflict of interest.

## Publisher’s Note

All claims expressed in this article are solely those of the authors and do not necessarily represent those of their affiliated organizations, or those of the publisher, the editors and the reviewers. Any product that may be evaluated in this article, or claim that may be made by its manufacturer, is not guaranteed or endorsed by the publisher.

## Footnotes

1
https://osf.io/n3a6s/

## References

[B1] Alter A. L. Kernochan J. Darley J. M. (2007). Transgression wrongfulness outweighs its harmfulness as a determinant of sentence severity. *Law Hum. Behav.* 31 319–335. 10.1007/s10979-006-9060-x 17268827

[B2] Andreescu V. Hughes T. (2020). Public opinion and the death penalty in Japan. *Punishm. Soc.* 22 573–595. 10.1177/1462474520915572

[B3] Berryessa C. M. (2018). The effects of psychiatric and “biological” labels on lay sentencing and punishment decisions. *J. Exp. Criminol.* 14 241–256. 10.1007/s11292-018-9322-x

[B4] Berryessa C. M. (2021). The potential influence of criminological rationales in considering childhood abuse as mitigating to sentencing. *Child Abuse Negl.* 111:104818. 10.1016/j.chiabu.2020.104818 33223305

[B5] Brubacher M. R. (2019). Third-party views of incarceration: justice, desistance, and offender reintegration. *Psychiatr. Psychol. Law* 26 693–705. 10.1080/13218719.2019.1618754 31984105 PMC6762182

[B6] Cañadas E. Rodríguez-Bailón R. Lupiáñez J. (2015). The effect of social categorization on trust decisions in a trust game paradigm. *Front. Psychol.* 6:1568. 10.3389/fpsyg.2015.01568 26528221 PMC4600900

[B7] Carlsmith K. M. (2006). The roles of retribution and utility in determining punishment. *J. Exp. Soc. Psychol.* 42 437–451. 10.1016/j.jesp.2005.06.007

[B8] Carlsmith K. M. (2008). On justifying punishment: the discrepancy between words and actions. *Soc. Justice Res.* 21 119–137.

[B9] Carlsmith K. M. Darley J. M. (2008). Psychological aspects of retributive justice. *Adv. Exp. Soc. Psychol.* 40 193–236. 10.1016/S0065-2601(07)00004-4

[B10] Carlsmith K. M. Darley J. M. Robinson P. H. (2002). Why do we punish? Deterrence and just deserts as motives for punishment. *J. Pers. Soc. Psychol.* 83 284–299. 10.1037/0022-3514.83.2.284 12150228

[B11] Cohen J. (1988). “Multiple Regression and Correlation Analysis,” in *Statistical Power Analysis for the Behavioral Sciences*, ed. Cohen J. (Hillsdale, NJ: Lawrence Erlbaum Associates), 407–465.

[B12] Cotton M. (2000). Back with a vengeance: the resilience of retribution as an articulated purpose of criminal punishment. *Am. Crim. Law Rev.* 37 1313–1362.

[B13] Crockett M. J. Özdemir Y. Fehr E. (2014). The value of vengeance and the demand for deterrence. *J. Exp. Psychol. Gen.* 143 2279–2286. 10.1037/xge0000018 25285429 PMC4242077

[B14] Daly K. (2002). Restorative justice: the real story. *Punishm. Soc.* 4 55–79. 10.1177/14624740222228464

[B15] Exum J. J. (2017). Should death be so different: sentencing purposes and capital jury decisions in an era of smart on crime sentencing reform. *Ark. Law Rev.* 70:227.

[B16] Gelfand M. J. Raver J. L. Nishii L. Leslie L. M. Lun J. Lim B. C. (2011). Differences between tight and loose cultures: a 33-nation study. *Science* 332 1100–1104. 10.1126/science.1197754 21617077

[B17] Gerber M. M. Jackson J. (2013). Retribution as revenge and retribution as just deserts. *Soc. Justice Res.* 26 61–80. 10.1007/s11211-012-0174-7

[B18] Goldman A. H. (1982). Toward a new theory of punishment. *Law Philos.* 1 57–76. 10.1007/BF00143146

[B19] Gollwitzer M. Bücklein K. (2007). Are “we” more punitive than “me”? Self-construal styles, justice-related attitudes, and punitive judgments. *Soc. Justice. Res.* 20 457–478. 10.1007/s11211-007-0051-y

[B20] Gromet D. M. Darley J. M. (2009). Punishment and beyond: achieving justice through the satisfaction of multiple goals. *Law Soc. Rev.* 43 1–38. 10.1111/j.1540-5893.2009.00365.x

[B21] Hollands J. G. Spence I. (1998). Judging proportion with graphs: the summation model. *Appl. Cogn. Psychol.* 12 173–190. 10.1002/(SICI)1099-0720(199804)12:2<173::AID-ACP499<3.0.CO;2-K

[B22] Hoskins Z. (2020). “Hybrid theories of punishment,” in *The Routledge Handbook of the Philosophy and Science of Punishment*, eds Waller B. Shaw E. Focquaert F. (New York, NY: Routledge), 37–48. 10.4324/9780429507212

[B23] Jiang S. Pilot R. Saito T. (2010b). Why Japanese support the death penalty? *Int. Crim. Justice Rev.* 20 302–316. 10.1177/1057567710373276

[B24] Jiang S. Lambert E. G. Wang J. Saito T. Pilot R. (2010a). Death penalty views in China, Japan and the US: an empirical comparison. *J. Crim. Justice* 38 862–869. 10.1016/j.jcrimjus.2010.06.001

[B25] Johnson D. T. (2020). “The death penalty and democracy,” in *The Culture of Capital Punishment in Japan. Palgrave Advances in Criminology and Criminal Justice in Asia*, ed. Johnson D. T. (Cham: Palgrave Pivot). 10.1007/978-3-030-32086-7_6

[B26] Keller L. B. Oswald M. E. Stucki I. Gollwitzer M. (2010). A closer look at an eye for an eye: laypersons’ punishment decisions are primarily driven by retributive motives. *Soc. Justice Res.* 23 99–116. 10.1007/s11211-010-0113-4

[B27] Kita M. Johnson D. T. (2014). Framing capital punishment in Japan: avoidance, ambivalence, and atonement. *Asian J. Criminol.* 9 221–240. 10.1007/s11417-014-9189-3

[B28] Kitayama S. Park H. Sevincer A. T. Karasawa M. Uskul A. K. (2009). A cultural task analysis of implicit independence: comparing North America, Western Europe, and East Asia. *J. Pers. Soc. Psychol.* 97 236–255. 10.1037/a0015999 19634973

[B29] Krosnick J. A. (1999). Survey research. *Annu. Rev. Psychol.* 50 537–567. 10.1146/annurev.psych.50.1.537 15012463

[B30] Lloyd A. Borrill J. (2020). Examining the effectiveness of restorative justice in reducing victims’ post-traumatic stress. *Psychol. Inj. Law* 13 77–89. 10.1007/s12207-019-09363-9

[B31] McCorkle R. C. (1993). Research note: punish and rehabilitate? Public attitudes toward six common crimes. *Crime Delinq.* 39 240–252. 10.1177/0011128793039002008

[B32] McFatter R. M. (1978). Sentencing strategies and justice: effects of punishment philosophy on sentencing decisions. *J. Pers. Soc. Psychol.* 36 1490–1500. 10.1037/0022-3514.36.12.1490

[B33] McKee I. R. Feather N. T. (2008). Revenge, retribution, and values: social attitudes and punitive sentencing. *Soc. Justice Res.* 21:138. 10.1007/s11211-008-0066-z

[B34] McMunigal K. C. (1998). Desert, utility, and minimum contacts: toward a mixed theory of personal jurisdiction. *Yale Law J.* 108 189–235. 10.2307/797473

[B35] Nagin D. S. (1998). “Deterrence and incapacitation,” in *The Handbook of Crime and Punishment*, ed. Tonry M. (New York, NY: Oxford University Press), 345–368.

[B36] Niang A. Leclerc C. Testé B. (2020). When punishment goals moderate and mediate the effect of clinical reports on the recidivism risk on prison sentences. *Psychiatry Psychol. Law* 1–15. 10.1080/13218719.2020.1805811 PMC9090404 35558147

[B37] Okimoto T. G. Wenzel M. Feather N. T. (2009). Beyond retribution: conceptualizing restorative justice and exploring its determinants. *Soc. Justice Res.* 22 156–180. 10.1007/s11211-009-0092-5

[B38] Orth U. (2003). Punishment goals of crime victims. *Law Hum. Behav.* 27 173–186. 10.1023/a:1022547213760 12733420

[B39] Oswald M. E. Hupfeld J. Klug S. C. Gabriel U. (2002). Lay-perspectives on criminal deviance, goals of punishment, and punitivity. *Soc. Justice Res.* 15 85–98. 10.1023/A:1019928721720

[B40] O’Toole M. J. Fondacaro M. R. (2017). When school-shooting media fuels a retributive public: an examination of psychological mediators. *Youth Violence Juv. Justice* 15 154–171. 10.1177/1541204015616664

[B41] Roberts J. V. Gebotys R. J. (1989). The purposes of sentencing: public support for competing aims. *Behav. Sci. Law* 7 387–402. 10.1002/bsl.2370070308

[B42] Robinson P. H. (1987). Hybrid principles for the distribution of criminal sanctions. *Northwest. Univ. Law Rev.* 82 19–42.

[B43] Robinson P. H. Darley J. M. (1995). *Justice, Liability, and Blame: Community Views and the Criminal Law.* New York, NY: Routledge. 10.4324/9780429039812

[B44] Rucker D. D. Polifroni M. Tetlock P. E. Scott A. L. (2004). On the assignment of punishment: the impact of general-societal threat and the moderating role of severity. *Pers. Soc. Psychol. Bull.* 30 673–684. 10.1177/0146167203262849 15155032

[B45] Shimizu H. (2016). An introduction to the statistical free software HAD: suggestions to improve teaching, learning and practice data analysis. *J. Media Inf. Commun.* 1 59–73.

[B46] Spiranovic C. A. Roberts L. D. Indermaur D. Warner K. Gelb K. Mackenzie G. (2012). Public preferences for sentencing purposes: what difference does offender age, criminal history and offence type make? *Criminol. Crim. Justice* 12 289–306. 10.1177/1748895811431847

[B47] Takikawa K. (2019). Is child abuse increasing? *Keishin Res. J.* 3 1–8. 10.24759/vetrdi.3.2_1

[B48] Templeton L. J. Hartnagel T. F. (2012). Causal attributions of crime and the public’s sentencing goals. *Can. J. Criminol. Crim. Justice* 54 45–65. 10.3138/cjccj.2010.E.29

[B49] Twardawski M. Tang K. T. Hilbig B. E. (2020). Is it all about retribution? The flexibility of punishment goals. *Soc. Justice Res.* 33 195–218. 10.1007/s11211-020-00352-x

[B50] Vidmar N. Miller D. T. (1980). Socialpsychological processes underlying attitudes toward legal punishment. *Law Soc. Rev.* 14 565–602.

[B51] Watamura E. Wakebe T. Maeda T. (2011). Can jurors free themselves from retributive objectives? *Psychol. Stud.* 56 232–240. 10.1007/s12646-011-0079-9

[B52] Weiner B. Graham S. Reyna C. (1997). An attributional examination of retributive versus utilitarian philosophies of punishment. *Soc. Justice Res.* 10 431–452. 10.1007/BF02683293

[B53] Witvliet C. V. Root Luna L. Worthington E. L. Jr. Tsang J. A. (2020). Apology and restitution: the psychophysiology of forgiveness after accountable relational repair responses. *Front. Psychol.* 11:284. 10.3389/fpsyg.2020.00284 32231606 PMC7082420

[B54] Yamamoto S. Maeder E. M. (2021). What’s in the box? Punishment and insanity in the Canadian jury deliberation room. *Front. Psychol.* 12:2442. 10.3389/fpsyg.2021.689128 34276516 PMC8277975

