# Supplementary material for: Justification of Sentencing Decisions: Development of a Ratio-Based Measure Tested on Child Neglect Cases
Source: Front Psychol. 2022 Jan 14;12:761536. doi: 10.3389/fpsyg.2021.761536 (PMC8796961; doi:10.3389/fpsyg.2021.761536)
Supplement: Supplementary file 1 [file Data_Sheet_1.DOCX]

Supplementary Material

# Supplementary Data

**New Scale**

(Note: Definitions of retribution, incapacitation, general deterrence, and rehabilitation derived from Berryessa [2018, pp. 245], for clarity and brevity).

In deciding on the punishment for this mother, how important are the following five items to you? Please enter the percentage of each so that the total is 100%.

***Retribution****: Retribution relies on the idea that for justice to be served, an offender deserves to be punished in a manner that is proportionate to the severity and moral heinousness of the committed crime.*

***Incapacitation****: Incapacitation aims to remove offenders from society to protect the public from future unlawful behavior.*

***General deterrence****: Deterrence attempts to prevent the future committal of crimes through the threat of future punishments that outweigh an individual’s motivation to commit future criminal acts.*

***Rehabilitation****: Rehabilitation seeks ways to actively reform and address the underlying reasons for an offender’s criminal behavior, so that an individual will not re-offend.*

***Precedent*** *(dummy): The sentence should be determined based on the sentencing decisions handed down from past abuse cases and judges’ opinions.*

**Sentencing**

*Please choose the one that is closest to your idea of punishment for this mother.*

(1) No punishment, (2) 1 day in prison, (3) 2 weeks in prison, (4) 2 months in prison, (5) 6 months in prison, (6) 1 year in prison, (7) 3 years in prison, (8) 7 years in prison, (9) 15 years in prison, (10) 30 years in prison, (11) life in prison

**Manipulation Check**

*Compared to other serious cases, the pain the child has suffered is much worse.*

*In order to prevent the mother from making the same mistake, it is important to keep her out of society.*

*I cannot help but wonder if this kind of child abuse is happening more often.*

*It is not entirely impossible that the mother can be rehabilitated.*

**Severe-damage scenario**

In April this year, Uemura Ikuto, who was two years old at the time, died of starvation in his apartment after not being fed for a long time. At the time of his rescue, he weighed 5.8 kg, less than half of the average weight; he was found to have swallowed pieces of paper and plastic, thus causing an intestinal blockage. The police arrested Ikuto’s mother, Riko (23), on charges of abandonment. The Child Guidance Center staff requested to visit the child repeatedly, but the mother refused to allow visits. There was information that she had divorced and become a single mother, but her whereabouts were unknown, and she could not be contacted. At the time of the incident, his mother had not returned home for at least six days, and although Ikuto was able to satisfy his hunger with a few seasonings, such as soy sauce and ginger paste, besides tap water, it is assumed that he collapsed and became immobile due to lack of nutrition. In his right hand, Ikuto was tightly holding a picture of his mother. When asked what her motive behind this abandonment was, the mother stated, “I wanted to spend time with my boyfriend. I thought it would be okay if he did not eat for a few days.”

**Moderate-damage scenario**

In April this year, Uemura Ikuto, who was two years old at the time, was found in a weakened state in his apartment after not being fed for a long time. At the time of his rescue, he weighed 5.8 kg, less than half of the average weight; he was found to have swallowed pieces of paper and plastic, and almost had an intestinal blockage but is fine now. The police arrested Ikuto’s mother, Riko (23), on charges of abandonment. The Child Guidance Center staff requested to visit the child repeatedly, but the mother refused to allow visits. There was information that she was divorced and became a single mother but her whereabouts were unknown and she could not be contacted. At the time of the incident, his mother had not returned home for at least 30 hours, and although Ikuto was able to satisfy his hunger with a few seasonings, such as soy sauce and ginger paste, besides tap water, it is assumed that he collapsed and became immobile due to a lack of nutrition. In his right hand, Ikuto was tightly holding a picture of his mother. When asked what her motive behind this abandonment was, the mother stated, “I lost my job due to the recession and had no money or energy. I thought it would be okay if he did not eat for a few days.”
